# Optimizing urban bike-sharing systems: a stochastic mathematical model for infrastructure planning

**DOI:** 10.1007/s10100-024-00950-z

**Published:** 2024-11-27

**Authors:** Seyedeh Asra Ahmadi, Peiman Ghasemi, Jan Fabian Ehmke

**Affiliations:** 1 Department of Logistics, Tourism and Service Management, German University of Technology in Oman, Muscat, Oman; 2 Department of Business Decisions and Analytics, University of Vienna, Kolingasse 14-16, 1090 Vienna, Austria; 3 Department of Business Decisions and Analytics, University of Vienna, Vienna, Austria

**Keywords:** Stochastic mathematical model, Chance constraint model, Bike-sharing systems, Peak and non-peak periods

## Abstract

This paper addresses the optimization of resource allocation and infrastructure planning in bike-sharing systems, particularly inspired by dynamic demand patterns as observed during the COVID-19 pandemic. We introduce a stochastic mathematical model that considers varying demand scenarios to enhance system performance and resource utilization. The research objectives are to fulfill the total travel demand across scenarios and compute the network's capacity to satisfy demand, thereby enhancing the system's efficiency and meeting users' diverse travel needs. The main contributions of this paper include presenting a stochastic mathematical model for bike-sharing station allocation and path network design, which optimizes resource allocation and infrastructure planning. Through a case study on the Vienna bike-sharing system, the model demonstrates practical applicability and effectiveness, offering insights for improving efficiency and service quality. The sensitivity analysis reveals that as costs for bicycle docks and station building increase, fulfilled demand decreases, emphasizing the crucial role of cost management in meeting demand efficiently.

## Introduction

During the spring of 2020, the Covid-19 pandemic led to a notable decline in mobility and motorized transport in Europe (Hintermann et al. 2023). This decrease prompted strict containment measures and reduced use of motor vehicles, especially in public transportation, due to infection risks (Schulte-Fischedick et al. 2021). Consequently, many people preferred using bicycles for personal transport to avoid infection (Jobe and Griffin 2021). Safety concerns, including infrastructure defects, were also raised. Bicycle-sharing systems emerged as a solution, offering shared bicycles to users without ownership (Chen et al. 2022). In response to these circumstances, cities enacted various measures such as establishing bike networks connected by “pop-up” bike lanes, widening sidewalks, and designating temporary shared spaces for activities promoting active mobility. These initiatives aimed to ensure adequate space for maintaining social distancing while facilitating the use of non-motorized modes of travel (Becker et al. 2022).

Users who wish to rent bikes from station-based bike-sharing systems are typically required to register as a basic step before receiving a card onto which they can deposit money to pay for their trips (Teixeira et al. 2023). This card acts as a bicycle key that unlocks bikes at the originating station. Users can take a bicycle from a station close to their starting point and park it at the nearest stop to their destination, where the bike is then parked, locked, and the rider is charged based on the duration of their journey. Fishman et al. (2012) presented that in most cases, short-term rentals are encouraged through price structures, often suggesting that the first 30 min are counted as free. Users can access information about station locations and bike availability at any time thanks to modern information technology. What distinguishes these bike-sharing systems from regular bicycle rentals is that riders do not need to return the bike to the exact same station they borrowed it from (Guo et al. 2024). As a result, they are ideal for one-way trips, specifically for traveling to the intended destination only (Peláez-Rodríguez et al. 2024). Furthermore, bike-sharing systems are not only considered an alternative solution for maximizing social distancing but are also regarded as one of the best transportation options for reaching remote places inaccessible to other means of transportation (Jin and Sui 2024). This makes them suitable for individuals who use public transportation but still need to walk to their final destination.

Beginning in mid-March 2020, Austria enforced comprehensive strategies aimed at curbing the spread of the COVID-19 outbreak. An analysis conducted by the Vienna Mobility Agency revealed that temporary infrastructure supporting sustainable transport, including pop-up bike lanes, played a pivotal role in fostering spaces for active mobility and yielded positive impacts on urban communities (Frey et al. 2023). Pop-up bike lanes offer a crucial solution to the pressing need for safer and more accessible cycling infrastructure in urban areas. Additionally, these temporary measures allow for quick implementation and community feedback, enabling city planners to design more effective and inclusive bike networks that meet the evolving needs of urban residents. Overall, pop-up bike lanes play a vital role in creating safer, more accessible, and more sustainable urban environments.

Designing a new bike-sharing network including bike lanes presents several advantages, foremost among them the ability to tailor the system to the city's specific needs and travel patterns, ultimately maximizing fulfilled travel demand. Furthermore, leveraging data-driven decision-making ensures that station locations and bike distribution are strategically placed to meet demand effectively. Scalability and flexibility in design allow for future growth and community engagement ensures that the network aligns with the preferences and priorities of the city's residents. Also, investing in the development of a new bike-sharing network in Vienna presents a unique opportunity to capitalize on the shifting transportation trends accelerated by the Covid-19 pandemic. A tailored bike-sharing system for Vienna, designed with strategic station locations connected by bike paths can fulfill travel demand by leveraging data-driven decision-making. The path-finding feature is crucial for enhancing user convenience and safety, as it provides optimized routes based on real-time data, guiding users along the most efficient and secure paths. It improves resource management by helping operators predict demand and optimize bike distribution, ensuring availability where and when needed most. It also enhances safety by promoting routes that are safer for cyclists, reducing the risk of accidents and encouraging more people to use the system. By incorporating data-driven decision-making and community engagement, the new system can better align with residents' needs and preferences, fostering greater acceptance and utilization.

So, this study introduces a stochastic mathematical model aimed at optimizing resource allocation and infrastructure planning in bike-sharing systems. It accounts for varying demand scenarios, particularly inspired by peak and non-peak periods of the COVID-19 pandemic, acknowledging the dynamic nature of demand patterns and their effects on system performance and resource usage. These scenarios represent different possible outcomes or realizations of travel demand, and they are used to capture the uncertainty in demand. Each scenario is associated with a probability weight, indicating the likelihood of its occurrence. The stochastic nature of travel demand in this paper is characterized by population proximity to stations, varied demand intensities, gravity distribution function, and route desirability estimation using a Logit model with carefully calibrated parameters. Through a case study on the Vienna bike-sharing system, the model's practical applicability and effectiveness in real-world settings are demonstrated, providing valuable insights for enhancing efficiency and service quality in urban bike-sharing networks.

This paper consists of six sections. In the first section, the introduction and statement of the problem are provided. The second and third sections contain the literature review and the mathematical model, respectively. Case study, computational results and conclusions are presented in Sects. 4, 5 and 6.

### Problem description

In the first step, a comprehensive network of bike-sharing systems is established, including routes, stations, dimensions between stations, and pop-up bike lanes connecting them. Moving on to the next step, which aims to determine the number of travel requests that the bike-sharing system will be able to meet, despite the different circumstances and time periods of each request during the day. This will help in finding robust solutions based on different demand scenarios. The bike-sharing network can be described by a graph G $(C,D),$ in which $C$ represents the locations of the proposed bike stations and $D$ represents the lanes connecting the stations.

“Normal stations” are strategically located throughout urban areas to cater to the local demand for bicycle trips. These stations serve as hubs where users can pick up and drop off bicycles conveniently. “Transit stations” are strategically located near major transportation hubs such as bus stops, metro stations, or train stations. These stations facilitate seamless integration between different modes of transportation, promoting intermodal mobility. Transit stations cater to commuters traveling longer distances, offering a convenient starting or ending point for their bike-sharing trips.

**Fig. 1:**
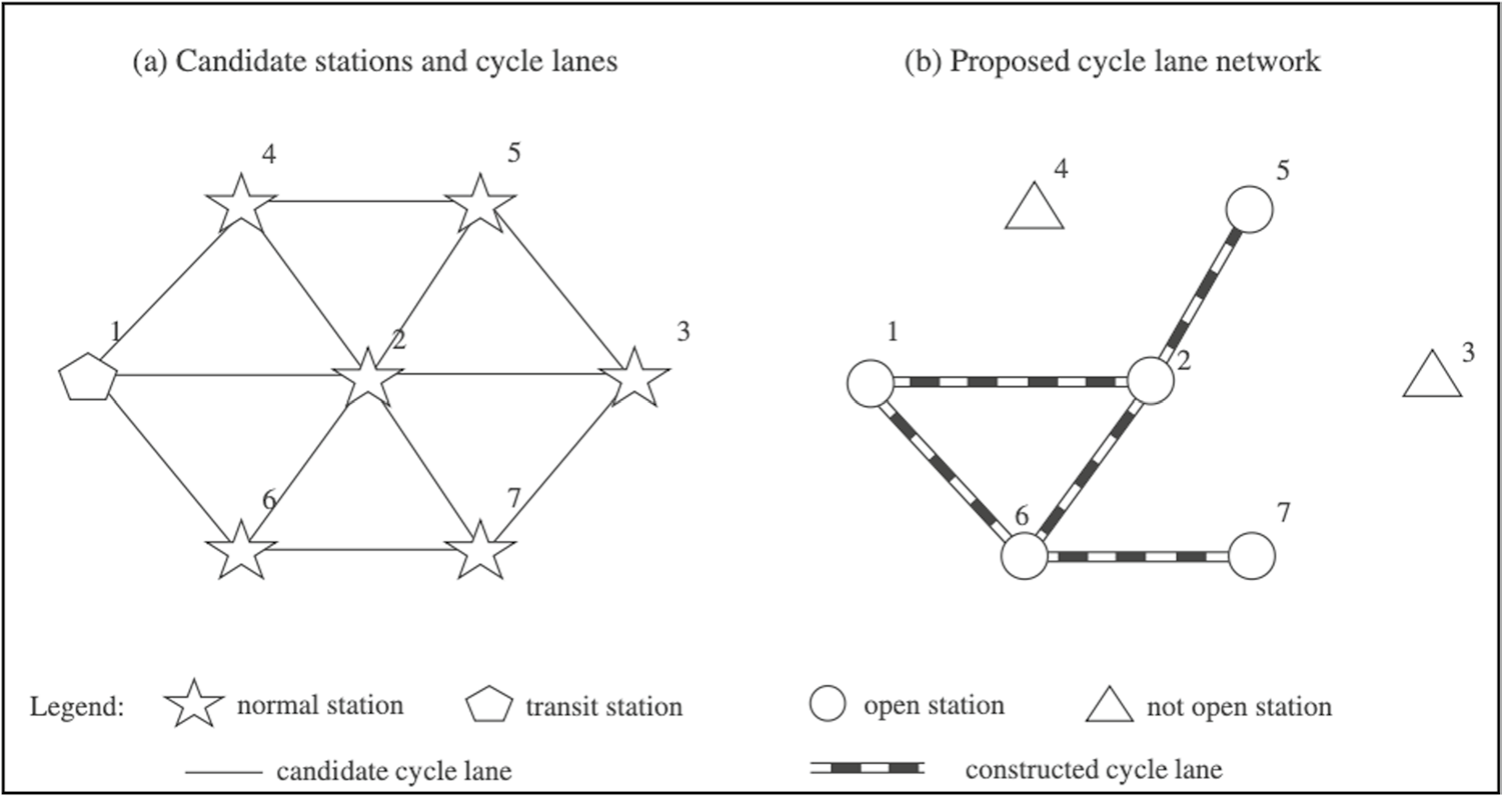
Illustration of bike-sharing stations and pop-up bike lanes network

Figure 1 shows the stations and pop-up lanes of the candidate bike-sharing network. As depicted, the stations are divided into two types: some are directly connected to the transit station, while others are not. For example, Stations 2 and 6 are directly connected to the transit station through corridors 1–2 and 1–6, just as Route 2–6 connects them directly. Stations like 7, however, are not directly connected to the transit station; their paths include 1-6-7 and 1-2-5, connecting them indirectly. The difference between stations directly connected to the transit station and those indirectly connected is that there is a station in the middle of the route that connects the preceding station to the transit station. Stations 3 and 4 are not connected due to lack of demand and limited budget. It is possible to directly connect station 2 as a starting point to destination point 6, and indirect paths such as paths 2-7-6 and 2-1-6 are also viable. Defining these routes ensures the presence of dedicated corridors that cover the entire course, as meeting demand requires interconnected dedicated pop-up lanes that span from the starting point to the intended destination. Figure 1 provides a clear example where demand can be met between Stations 5 and 6 due to the presence of dedicated lanes, while demand between Stations 4 and 6 cannot be satisfied. Although longer and indirect paths partially meet demand, the study assumes that satisfying demand with the shortest possible path is more satisfactory to users.

Our research objectives aim to fulfill the expected total travel demand across various scenarios and compute the total travel demand that the constructed bicycle network can satisfy for each scenario. These objectives address research questions focused on the allocation of bicycle docks to stations and strategies to meet fulfilled travel demand. By optimizing dock allocation and devising effective strategies, the study aims to enhance the efficiency and effectiveness of the bicycle-sharing system in meeting the diverse travel demands of users.

## Literature review

The literature review is presented in four parts. The first part deals with uncertainty in the bike-sharing problem. Bike sharing during the pandemic is examined in the second part. Next, in the third part, the bike station network design problem is discussed. Finally, in the last part, contributions are outlined.

### Uncertainty in bike-sharing optimization

Chen et al (2023) introduced a unique target-based stochastic model aimed at improving both efficiency and fairness in bike-sharing systems by considering uncertain demand. They utilize a risk measure for user dissatisfaction and a lexicographic order method to ensure equal service across different areas. The model cleverly incorporates external factors such as weather and weekends, creating a robust optimization framework that can be efficiently solved. Real-world data tests reveal that this model outperforms existing ones in providing efficient and equitable services. Interestingly, setting less ambitious targets might lead to better results. Wang et al. (2023) recognize the importance of addressing the uncertainty inherent in demand forecasting for bike-sharing services. They aim to align the supply and demand of bikes more effectively, focusing on optimal distribution. Their innovative strategy involves using spatiotemporal big data to understand user behaviour and identify key areas for bike deployment. The core of their model is the NSGA-II algorithm, which aims to lower dispatching costs while increasing bike usage. Hua et al. (2022) aim to minimize the number of bikes required in bike-sharing systems to meet unpredictable future demand, focusing primarily on reducing the fleet size. The methodology discussed is a quantitative approach that utilizes historical records to better arrange fleet assets; this method was implemented in Nanjing, China. The findings demonstrate a significant 44.6% reduction in the number of fleet vehicles while successfully fulfilling 96.8% of the requested trips, thus efficiently achieving the goal outlined in the study. Fu et al. (2022) introduce a novel approach to improve bike-sharing systems with a two-stage robust optimization model. This model is designed to boost daily earnings and effectively manage bike stations and inventories. It specifically tackles uncertainties in demand and the need for station rebalancing. The model's practical application in real-life settings has shown significant enhancements in bike-sharing operations, proving its value in enhancing these services. Jin et al. (2020) aim to optimize the distribution of bike-sharing stations and the construction of path networks while accounting for their associated costs. The goal of the paper is to maximize the predicted travel demand that can be met by the proposed network. The paper's contribution is a two-stage stochastic programming model that generates robust solutions by considering various demand scenarios. A case study conducted in Montevideo, Uruguay, demonstrated that the strategy used maximized demand coverage and created a densely connected bicycle infrastructure system.

### Bike sharing systems during pandemics

Teixeira and Lopes (2020) examine how bike-sharing and subway use in New York City were impacted by the COVID-19 pandemic, particularly analysing Citi Bike's performance. They find that bike-sharing systems showed more resilience compared to subways, with a smaller decrease in users and increased trip lengths. This study points to a possible shift from subway to bike-sharing as a transport mode during the pandemic, emphasizing the vital role bike-sharing can play in urban transport during challenging times. Rahimi et al. (2021) aim to study the perceived risk involved in the use of shared mobility services with reference to public transport and ride-sharing amidst the COVID-19 epidemic. The purpose is to minimize the difficulties of usage, for which knowledge of such perceived risks is required. The importance of this study lies in the analysis of survey data from the Chicago metropolitan area using the bivariate ordered model. The objective is to identify specific socio-demographic and external factors that influence the perceived level of risk. The results suggest that participants with low income and those living in regions with confirmed coronavirus cases experience a higher perception of risk. Chibwe et al. (2021) emphasize the significance of understanding demand variance in bicycle-sharing systems to enhance system development. Their study focuses specifically on the City of London and analyses the correlation between bicycle-sharing system demand and regional unemployment rates. Additionally, the study examines the impact of other variables such as weather fluctuations and differences between working days and holidays on demand levels. Utilizing a generalized negative binomial model, the research aims to comprehend demand uncertainty and its variations, offering insights to improve system management and efficiency. Karatas et al. (2022) propose addressing the problems associated with transportation and location planning under pandemic conditions. The aim is primarily to reduce the effects pandemics have on transportation and logistics networks. The contribution of the paper lies in a new classification of decision problems to address these issues concerning logistics, mobility, and waste management. The results demonstrate the successful utilization of proposed model in reducing interruptions caused by the pandemic. Thomas et al. (2024) present a heuristic simulation-based method for optimizing bike-sharing networks based on user activity in post pandemic. One contribution of their model was considering the Distance-Willingness-Reward matrix for determining motivators of bike users. The results and sensitivity analysis show that the proposed network service level enhanced, even with smaller user willingness levels.

### Bike stations network design problem

Song et al. (2024) craft a dual-layered model to optimize the placement of stations in bike-sharing networks, accounting for both standard and electric bikes. They use a Genetic Algorithm for the higher-level decisions and the rolling horizon method for more detailed lower-level planning, enhancing it with sophisticated decomposition and simplex methods. The practicality and effectiveness of their model in determining station locations are illustrated through real-world numerical cases, shedding light on ways to improve bike-sharing setups. Jiménez & Soriguera (2024) aim to increase the efficiency of bike-sharing repositioning using a reactive and holographic approach. The paper seeks to minimize the penalties incurred by users when bikes or docking stations are unavailable. The authors propose a method for assigning relocation tasks to vehicles in real-time, which does not require precise demand forecasting. The effectiveness of this implementation strategy is demonstrated in a study conducted in Barcelona, Spain, showing improvements in system performance. Jin and Sui (2024) aim to assess equity in bike-sharing systems across 73 cities in the United States. Their primary objective is to minimize disparities in bike-sharing coverage among deprived neighborhoods. The study applies a generalized additive mixed model to evaluate the fairness of bike-sharing in relation to different social factors. The findings highlight notable differences, particularly in communities characterized by higher levels of deprivation and minority populations. A case study conducted in various U.S. locations reveals that, although populations without vehicles tend to experience improved accessibility, other marginalized groups remain underserved. Walker and Kwon (2024) propose a model aimed at optimizing daily bike inventory management in bike-sharing networks. Their objective is to find the best inventory levels for each station to reduce costs related to moving bikes and unfulfilled user demands. By applying their model to real data from Houston's Bicycle, they successfully show that their approach can significantly improve the operational efficiency and user experience of bike-sharing systems. Cheng et al. (2022) develop a bi-level model aimed at enhancing bike-sharing infrastructure. They focused on reducing both the costs of construction and the travel times for cyclists and drivers. Their unique contribution is a sequence-based hyper-heuristic algorithm, which incorporates a Hidden Markov Model for more effective heuristic choices. The model's efficiency and practicality in improving bike-sharing systems and encouraging cycling are evidenced through various numerical examples.

Xu et al. (2020) highlight the necessity of establishing a sustainable bike-sharing system, emphasizing the crucial role of comprehensive network connectivity and accessibility. Previous studies predominantly focused on supplier allocation within the system, neglecting infrastructure investments for cycling path allocation. The authors advocate for city and street planners to forecast bicycle demand and invest in dedicated bike paths, underscoring the importance of proactive planning to ensure the effectiveness and longevity of bike-sharing systems.

### Contributions

The existing literature presents significant research contributions in bike-sharing systems, yet several gaps remain. While the stochastic model addresses demand variations, the integration of real-time data analytics for adaptive decision-making has not been considered in other research, limiting the system's ability to respond to real-time changes. Additionally, long-term behavioral changes driven by the COVID-19 pandemic have been ignored in other studies, leaving potential shifts in demand patterns unexplored. Finally, the other models do not consider the integration of scenario-based multi-period bike-sharing systems during pandemic in real case studies (see Table 1).

**Table 1:**
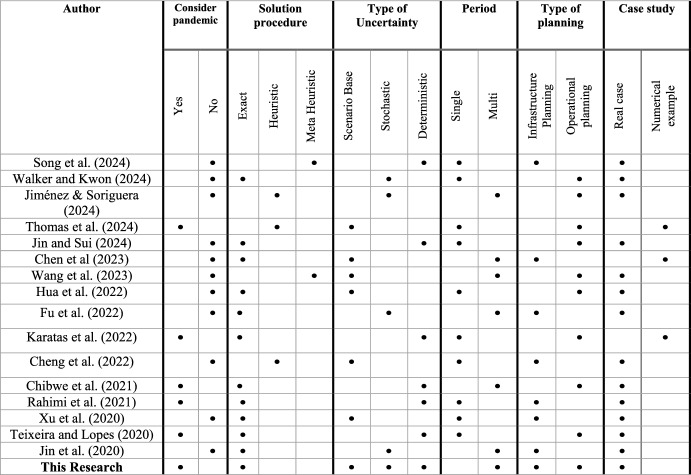
Literature on the bike-sharing systems

The existing literature presents significant research contributions in bike-sharing systems, yet several gaps remain. While the stochastic model addresses demand variations, the integration of real-time data analytics for adaptive decision-making has not been considered in other research, limiting the system's ability to respond to real-time changes. Additionally, long-term behavioral changes driven by the COVID-19 pandemic have been ignored in other studies, leaving potential shifts in demand patterns unexplored. Finally, the other models do not consider the integration of scenario-based bike-sharing systems in a pandemic situation in real case studies.

Based on the research gaps, the contributions of our paper can be summarized as follows:

- We present a stochastic, scenario-based and multi-period mathematical model for bike-sharing stations allocation and path network design, providing a comprehensive framework for optimizing resource allocation and infrastructure planning in bike-sharing systems.
- We consider demand scenarios during peak and non-peak periods of the COVID-19 pandemic, addressing the dynamic nature of demand patterns and their impact on system performance and resource utilization.
- We apply the proposed model to a real case study in the Vienna bike-sharing system, demonstrating the practical applicability and effectiveness of the model in real-world settings, and offering insights for improving system efficiency and service quality in urban bike-sharing networks.

## Mathematical model

This section includes a description of the problem formulation for each of sets and indexes, parameters and decision variables. The assumptions are as follows:

- The model assumes that the entire population residing within a 300-m radius of each station contributes to the travel demand estimation.
- The percentage of bicycle demand relative to the entire population is used as a measure of bicycle demand intensity.
- The use of a gravity distribution function assumes that travel demand between origin and destination points follows a pattern similar to gravitational forces, where demand decreases with increasing distance and increases with increasing population size.
- Station cost is the cost associated with establishing a bike station. It includes the infrastructure needed to set up the station, such as land acquisition and construction. Dock cost refers to the cost of installing individual docking points within a station.
- The model assumes that there will be no shortage of bikes at stations.

The model is based on Jin et al. (2020). In particular, we used the first stage of the mathematical model and added a pandemic scenario including a stochastic chance constraint.

### Sets and indices

$N$: Group of nodes which stand for nominated bicycle stations, ($a,b)\in N$

$L$: Links illustrating the direct paths between each two adjacent bike sharing stations.

$E$: Pair’s group of origin–destination for travel demand by bike.

${D}_{e}$: Possible paths for the origin–destination pair $e \in E$ in the bicycle sharing network.

$$\Omega$$: Demand scenario.

$T$: Time steps for one hour.

### Parameters

$\xi$**:** Pair of Origin–Destination $e \in E$ travel amount, at period of time $t \in T$ and under the scenario of demand $\xi \in\Omega$.

${C}_{a}$: Bikes station building cost.

${C}_{ab}$: Bikes paths on link $(a,b)$ building cost.

${C}_{0}$: Costs for docks of bikes.

$${d}_{ab}$$: Link $$(a,b)\in N$$ distance.

$K$: Available total budget.

${o}_{e}$: Pair of Origin–Destination $e \in E$ origin node.

${u}_{e}$: Pair of Origin–Destination $e \in E$ destination node.

${Q}_{max}$: Bicycle docks maximum number in each station.

${W}_{ed}$: number of links on path $d\in {D}_{e}$ of Origin–Destination pair $e \in E$.

${b}_{ab}^{ed}$: Binary coefficient expresses the relationship between the path $d\in {D}_{e}$ of origin–Destination pair $m \in M$ and link $(a,b)$, as it equals 1 if link $(a,b)$ is part of Origin–Destination pair $e \in E$, otherwise it is equal to 0.

${\beta }_{ed}$: The percentage of travel demand that can be satisfied for the pair of Origin–Destination $e \in E$ by path $d \in {D}_{e}$.

$H(\xi )$: The weight of demand scenario $\xi \in\Omega$.

$$M$$: large Number.

${\widetilde{T}}_{et} \left(\xi \right)$: Total travel demand at period $t \in T$ in Origin–Destination pair $e\in E$

### Decision variables

${F}_{a}\in \{\text{0,1}\}$: In case that bicycle station $a \in N$ is selected then it is equal to 1; while it is equal to 0 otherwise.

${G}_{ab }\in \{\text{0,1}\}$: In case that the lane of bicycle is created on link $\left(a,b\right)\in L$ then it equals 1; 0 otherwise.

${z}_{a}$: Bicycle-docks number in station $a \in N$.

${\delta }_{ed }\in \{\text{0,1}\}$: In case that the lanes of bikes are created on path $d \in {D}_{e}$ links for the pair of Origin–Destination $e \in E$ then it is equal to 1; 0 otherwise.

${R}_{et} (\xi )$: Origin–Destination $e \in E$ travel demand satisfied at time period $t \in T$.

### Objective function and constraints

1 $$Maximize \mathop \sum \limits_{e \in E} \mathop \sum \limits_{t \in T} H\left( \xi \right)R_{et} \left( \xi \right)$$

2 $$F_{a} \ge G_{{ab}}\, \forall \;\left( {a,b} \right) \in L$$

3 $$F_{b} \ge G_{{ab}}\, \forall \;\left( {a,b} \right) \in L$$

4 $$z_{a} \le Q_{{max}} \;F_{a} \;\,\forall a \in N$$

5 $$\sum\limits_{{(a,b) \in L}} {b_{{ab}}^{{ed}} } G_{{ab}} - W_{{ed}} \ge M\left( {\delta _{{ed}} - 1} \right)\;\,\forall e \in E,\;\forall d \in D_{e}$$

6 $$\sum\limits_{{(a,b) \in L}} {b_{{ab}}^{{ed}} } G_{{ab}} - W_{{ed}} + \varepsilon \le M.\delta _{{ed}} \;\,\forall e \in E,\;\forall d \in D_{e}$$

7 $$\delta _{{ed}} \le \sum\limits_{{\left( {a,b} \right) \in L}} {b_{{ab}}^{{ed}} } G_{{ab}} /W_{{ed}} \;\,\forall e \in E,\;\forall d \in D_{e}$$

8 $$\sum_{a\in N}{C}_{a}{F}_{a} +\sum_{(a,b)}{C}_{ab}{G}_{ab } + {C}_{0}\sum_{a}{z}_{a}\le K$$

9 $${R}_{et} \left(\xi \right)\le {\widetilde{T}}_{et} \left(\xi \right)\, \forall e\in E,\forall t\in T,\forall \xi \in\Omega$$

10 $$-{z}_{a}\le \sum_{\left(e|{o}_{e}=a\right)}{R}_{et} \left(\xi \right)-\sum_{\left(e|{u}_{e}=a\right)}{R}_{e\left(t-1\right)}\le {z}_{a} \forall a\in N,\, \forall t\in T, \forall \xi \in\Omega$$

11 $${F}_{a}\in \left\{\text{0,1}\right\}\, \forall a\in N$$

12 $${G}_{ab }\in \left\{\text{0,1}\right\}\, \forall (a,b)\in L$$

13 $${z}_{a} ,{R}_{et} \left(\xi \right)\in integer\, \forall a\in N$$

14 $${\delta }_{ed }\in \left\{\text{0,1}\right\} \,\forall e \in D,\forall d\in {D}_{e}$$

The objective function (1) aims to maximize the fulfilled demand of travel ${R}_{et} (\xi )$ across all different demand scenarios. It encompasses decisions related to the construction of bike lanes ${G}_{ab } and {\delta }_{ed}$, as well as the determination of station locations ${F}_{a} and {z}_{a}$. Constraints (2) and (3) establish the connection between ${F}_{a} and {G}_{ab }$ and ensure that the corresponding station is determined once a link $(a,b)$ is built. Constraint (4) is crucial to prevent exceeding the maximum number of docks. The relationship between ${\delta }_{ed } and {G}_{ab}$ is depicted by constraints (5), (6), and (7): when all links on the path are covered by bike lanes, ${\delta }_{ab}$ equals 1; otherwise, it equals 0. Constraint (8) is essential to ensure that the cost of building bike-sharing lanes, constructing bike berths, and locating bike stations remains within the available budget. Constraint (9) ensures that the demand for travel does not surpass the total demand for travel during specific periods and for each origin–destination pair. Constraint (10) ensures that the number of requests that can be answered at the moment must always be smaller than the inventory for the current period. Regarding the scenario where bikes arrive and leave at the same time instant, potentially exceeding available docks and demands, our model works as follows. We account for this by incorporating the balance of bicycles from the previous period into the current system's inventory. Specifically, if a bicycle reaches its destination in the previous period, it is added to the system inventory for the new period, as represented by $\sum_{\left(e|{u}_{e}=a\right)}{R}_{e\left(t-1\right)}$. This approach ensures that any handoff between users, where a bike is directly transferred without occupying a dock, is reflected in the model. By adding the previous period's balance to the current inventory, we capture scenarios where multiple requests are satisfied without exceeding the system's capacity. Decision variables are defined by constraints (11) to (14).

### Stochastic chance constraint programming

We solve the above model with Stochastic Chance Constraint Programming (SCCP), which has been proposed previously by Charnes and Cooper (1959) and has been used by, e.g., Jiang and Guan 2016 and Kamran et al. 2023. First, we assume that ~ is the symbol of an uncertain parameter and *n* is the number of objective functions that we have in our mathematical model. Also, we assume that parameters ${a}_{mn}, {h}_{m}, {c}_{nl}$ are defined as stochastic parameter, which are used in Eq. (15) through Eq. (18). Accordingly, the uncertain model is shown below as follows:

15 $$\min f_{h} = E\left( {\sum\limits_{{j = 1}}^{n} {b_{{rj}}^{\sim } } v_{j} \ge u_{i}^{\sim } } \right)\quad \begin{array}{*{20}l} {h = 1, \ldots ,K} \hfill \\ {i = 1,2, \ldots ,m} \hfill \\ \end{array}$$

16 $$p\left({\sum }_{j=1}^{n} {g}_{ij}^{\sim }{v}_{j}\ge {u}_{i}^{\sim }\right)\ge {\alpha }_{i}\quad i=\text{1,2},\dots ,m$$

17 $$v=\left({v}_{1},\dots ,{v}_{n}\right)$$

18 $$v\ge 0$$

where ${b}_{rj}^{\sim }$  represents the benefit ratio of the $jth$ decision variable in the $rth$ objective function. Additionally, other parameters such as ${g}_{ij}^{\sim }$ represent the technology coefficient (indicates the relationship between resources and decision variables). Also, ${u}_{i}^{\sim }$ is the right-hand side of the $mth$ constraint, and ${v}_{j}$ is also a decision variable.

For more information on this transformation, see Reza-Pour and Khalili-Damghani, (2017) and Appendix A. Based on the proposed mathematical model, the chance constraint model at the α% level for the constraint 9 is defined as follows:

19 $${R}_{et} (\xi )\le (E\left({T}_{et} \left(\xi \right)\right)+{\varphi }^{-1}\left(1-{\alpha }_{i}\right)\sqrt{var\left({T}_{et} \left(\xi \right)\right)} \forall e\in E,\forall t\in T,\forall \xi \in\Omega$$

with ${R}_{et} (\xi )$ as the travel demand satisfied at time period $t \in T$, $E\left({T}_{et} \left(\xi \right)\right)$ as the expected value of total travel demand, and $var\left({T}_{et} \left(\xi \right)\right)$ as the variance of total travel demand at the α% level for the constraint.

## Case description and parameter setting

In April 2020, the City of Vienna implemented initiatives to expand space for pedestrians and cyclists amid the COVID-19 crisis, aiming to uphold social distancing and health guidelines. These efforts comprised two key components:

- Implementation of temporary “shared space” streets: This entailed repurposing sections of streets to accommodate pedestrians and cyclists, providing communal areas for public use while facilitating adherence to social distancing protocols.
- Introduction of “pop-up cycling lanes”: This involved establishing designated lanes for cyclists in specific areas of the city, enhancing their ease and safety of travel and promoting the advantages of bicycle transportation.

These measures were enacted to enhance the safety and convenience of pedestrians and cyclists during the COVID-19 pandemic, enabling smoother movement in public spaces while facilitating compliance with relevant health guidelines.

To check the proposed mathematical model, a case study of bicycle sharing in the city of Vienna is conducted as follows. First, an interview was conducted with Martin Blum, who is the head of the Mobility Agency and the official representative of Cycling Affairs in Vienna. The remaining information was obtained from emails with bike-sharing operators, academic articles and publications, and infrastructure data from open street maps. The idea of bicycle sharing in the city of Vienna was first formed in 1991 when the company Veloce tried to implement this idea, but it was not supported by the government. Following Wiener Stadtrad by Siems & Klein KG, this concept was expanded in 1997 (Dechant, 2013). Viennabike, the first official bicycle sharing system of the Viennabike company, was established in 2002, and immediately Citybike Wien took its place, which is still active.

In this research, the cost of the stations is considered to be equal to 40,000 units per station, and the cost of building the tracks is considered to be equal to 2000 units per 100 m. The cost of each bicycle dock is considered to be equal to 40 units, and the available budget is equal to 600,000 units. Additionally, 23 stations along with 23 cycling paths, which are 29 km long in total, are considered for experiments. Demand scenarios are based on two criteria: travel intensity and time period (see Table 2).

**Table 2:** Demand scenarios

Demand	Number	Explanation
Period	2	Peak, non-peak
Intensity	3	5%, 10%, 15%

Two-time patterns, including the peak of Covid-19 and non-peak times, have been considered. Travel demand is also calculated based on the population within 300 m of each station. The intensity of demand is also based on a percentage of the total demand.

A gravity distribution function is used to estimate the demand. This distribution function is inspired by Newton’s law of gravitation, where the attraction (or flow) between two points depends on the "mass" (in this case, the demand intensity) and the "distance" (or travel cost) between them. The demand intensity could be represented as the number of people or goods that need to be transported between two locations. The travel cost is often determined by the distance or the time required to travel between these locations. Also, three demand intensities of 5%, 10%, and 15% are considered. Therefore, six scenarios can be imagined for the proposed model. The ${\beta }_{ed}$ parameter should also be carefully calibrated because it plays an important role in the proposed model. To achieve this, the model uses a Logit model, which is a statistical model often used in transportation to estimate the probability of choosing one route over others. The Logit model compares the desirability of each route with all other possible routes, including the shortest route. For the sake of clarity, we simplify and calculate the shorter path as 100% and the inverse ratio of the path length as Eq. (20):

20 $$\beta _{{ed}} = \frac{{Min_{{k^{\prime } }} \sum\limits_{{(a,b)}} {d_{{ab}} } b_{{ab}}^{{ed^{\prime } }} }}{{\sum\limits_{{(a,b)}} {d_{{ab}} } b_{{ab}}^{{ed}} }}$$

where ${d}_{ab}$ represents the distance or cost between two points $a$ and $b$ in the network and ${b}_{ab}^{ed}$ and $b_{{ab}}^{{ed^{\prime } }}$ refer to some specific properties of the links (or segments) between nodes $a$ and $b$ in the transportation network. The superscripts $ed$ and $ed{\prime}$ could correspond to different scenarios or demand intensities. Also, the numerator, $Min_{{k^{\prime } }} \sum\limits_{{(a,b)}} {d_{{ab}} } b_{{ab}}^{{ed^{\prime } }}$, represents the total cost of the shortest route (which is simplified to 100%). Furthermore, the denominator, $\sum_{(a,b)}{d}_{ab}{b}_{ab}^{ed}$, is the total cost of a candidate route (different from the shortest path).

**Fig. 2:**
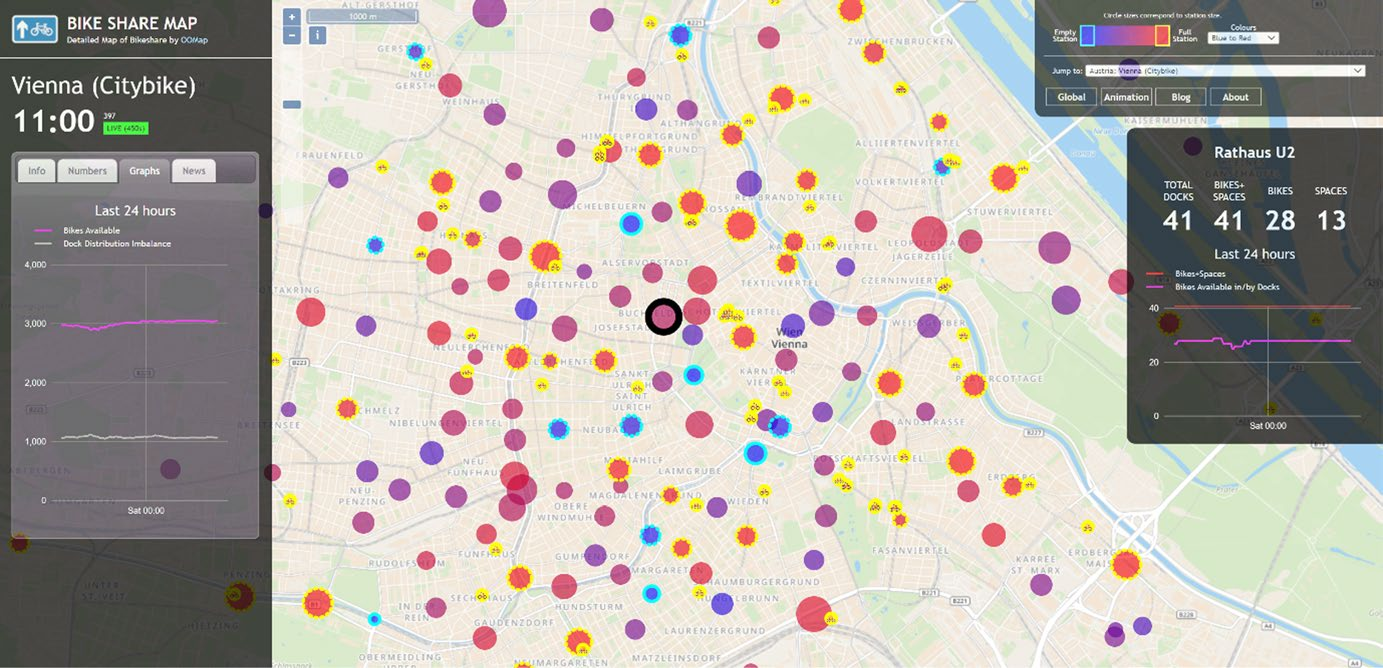
Case study map (https://bikesharemap.com/vienna/#/)

The Bike Share Map showcases the docking station locations associated with Vienna's bicycle sharing systems. Each station is depicted by a circle with its size and colour indicating the number and size of bicycles present at the moment. The maps typically refresh every few minutes, and there is also a version that replays the colour and size alterations from the past 48 h (see Fig. 2).

Also, the website https://www.wien.gv.at/stadtplan/ was used to calculate the distances and durations between the candidate stations, as well as the actual parameter instances shown in Appendix B.

## Computational results

In this section, computational results related to the proposed model are presented. The model performance was validated using GAMS 42.1.0 with the CPLEX 11.0 solver on an Intel(R) Core(TM) i7 CPU running at 1.60 GHz with 16 GB RAM.

This section is divided into three parts. In the first part, the results of the research, including the comparison of peak Covid-19 and non-peak Covid-19 scenarios, are presented. In the second part, a sensitivity analysis is performed on the objective functions and important parameters of the model. In the third part, a sensitivity analysis is performed on the model CPU time.

### Overall results

**Fig. 3:**
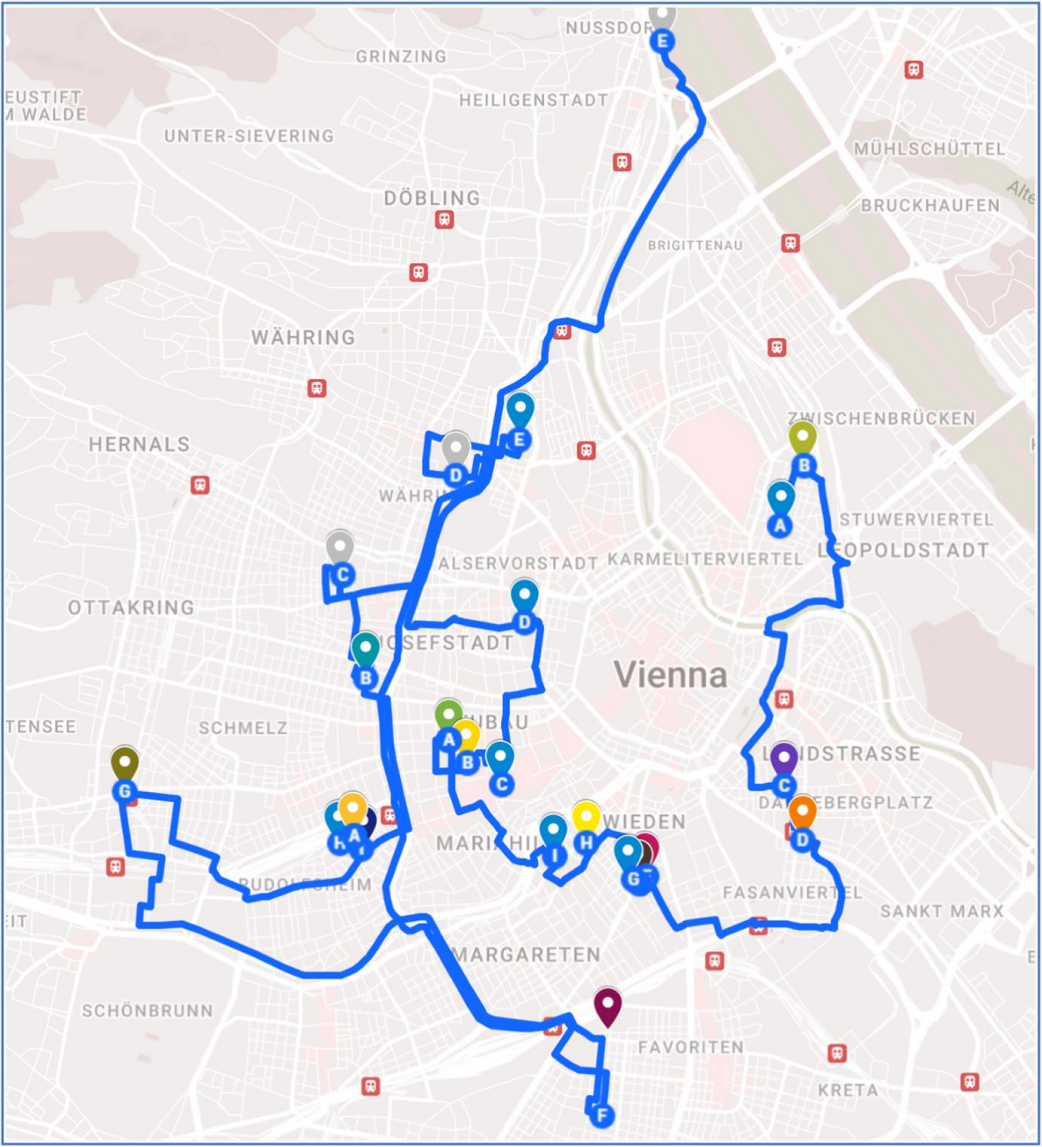
Proposed network configuration during pandemic

Figure 3 illustrates the bicycle stations that have been established in Vienna for the pandemic scenario. A total of 23 stations have been set up. The proposed network demonstrates excellent connectivity, with the travel paths between stations closely approximating the shortest possible routes. Additionally, the bicycle lanes form a loop, providing users with multiple route options.

Table 3 shows the number of docks at established stations. For example, 15 docks have been established at Pazmanitengasse station, and 24 docks at Alliiertestraße station.

**Table 3:** Number of established docks

District, Stations	Dock number	District, Stations	Dock number
2, Pazmanitengasse, Vienna	23	8, Florianigasse, Vienna	22
2, Alliiertestraße, Vienna	10	9, Sobieskigasse, Vienna	22
3, Rechte Bahngasse, Vienna	27	10, Fernkorngasse, Vienna	12
3, Schützengasse, Vienna	9	14/15, Meiselstraße, Vienna	22
4, Graf-Starhemberg-Gasse, Vienna	16	15, Rosinagasse, Vienna	18
4, Schaumburgergasse, Vienna	16	15, Gasgasse, Vienna	32
4, Große Neugasse, Vienna	18	15, Zwölfergasse, Vienna	18
4/5, Kettenbrückengasse, Vienna	14	16, Hasnerstraße, Vienna	15
5, Rüdigergasse, Vienna	15	17, Kalvarienberggasse, Vienna	14
7, Kandlgasse, Vienna	15	18, Schopenhauerstraße, Vienna	16
7, Hermanngasse, Vienna	15	20, Brigittenauer Sporn, Vienna	17
7, Zollergasse, Vienna	18		

**Fig. 4:**
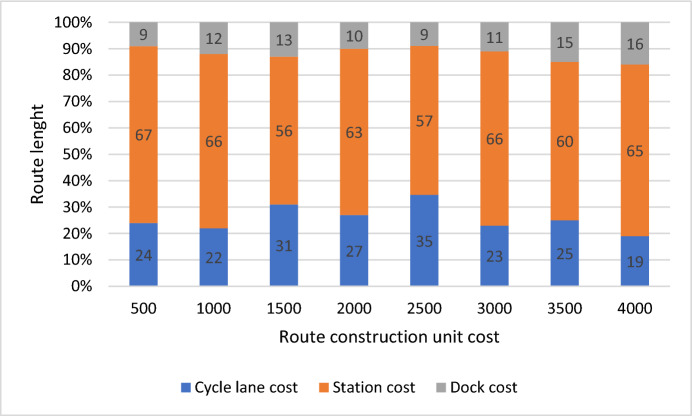
Distribution of budget based on route costs

Figure 4 provides a comprehensive breakdown of the distribution of costs associated with route length, encompassing crucial components such as cycle lane expenses, station establishment costs, and dock investments. The vertical axis represents route length, while the horizontal axis represents route contraction costs, which include costs for cycle lanes, stations, and docks. The data clearly illustrates a significant allocation of resources towards station establishment, representing a substantial 62.5% of the overall budget. This emphasizes the pivotal role of stations within the infrastructure framework, underlining their significance in facilitating efficient transportation systems. Furthermore, the analysis reveals that 25.75% of the total expenses are designated for the establishment and maintenance of cycle lanes. This allocation underscores the commitment to promoting sustainable and eco-friendly modes of transportation, aligning with contemporary urban planning initiatives aimed at enhancing cycling infrastructure. Lastly, the remaining portion of the budget is directed towards dock investments, indicating a strategic focus on providing convenient access points for users of the transportation network. This holistic approach to resource allocation underscores a balanced investment strategy that prioritizes essential elements of infrastructure development, ensuring the seamless integration of cycling amenities within the urban landscape.

**Fig. 5:**
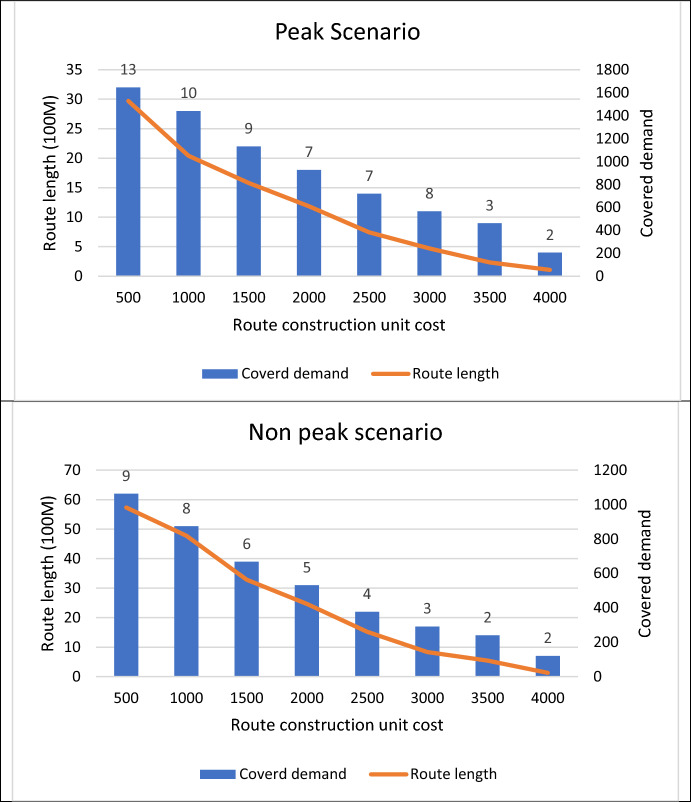
Route length and number of stations based on demand coverage and route costs

Figure 5 presents the results of solving the proposed mathematical model, which is based on route length, number of stations, demand coverage, and route costs. Additionally, the labels in the figure correspond to the number of established stations with the available specifications. According to the results, the length of the routes in the designed cycling network increases as the cost of constructing the routes decreases. This implies that, in this scenario, the demand for cycling travel is being met. The results indicate a crucial interplay between route length, cost, and demand coverage in designing a cycling network. As the cost of constructing routes decreases, there is a corresponding increase in route length, suggesting a positive correlation between investment in infrastructure and meeting the demand for cycling travel. This insight underscores the importance of strategic investment in infrastructure to effectively cater to the needs of cyclists while optimizing costs, ultimately enhancing the accessibility and attractiveness of cycling as a mode of transportation.

In the peak scenario, due to high demand, more stations are established within the cycling network. This indicates a proactive response to accommodate the higher volume of cyclists during peak periods. The analysis reveals that in the peak scenario, the length of cycling routes increases. This extension of route lengths suggests a strategic effort to meet the heightened demand for cycling travel by providing longer and potentially more interconnected routes.

**Fig. 6:**
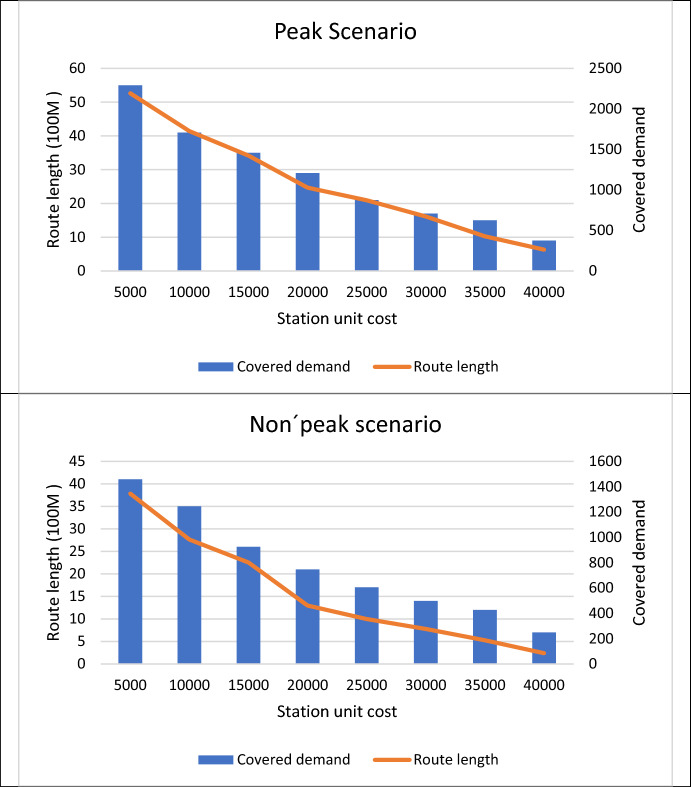
Route length based on demand coverage and station costs

Figure 6 presents how adjusting station costs impacts route length and demand coverage. By reducing station unit costs, longer routes can be established, leading to a broader coverage of demand. This not only enhances the efficiency of the cycling network but also promotes its utilization by a wider population. Therefore, these findings emphasize the importance of strategic planning and investment in infrastructure to create a robust cycling network that effectively addresses the evolving demands of urban mobility while ensuring cost-effectiveness. Such initiatives not only contribute to reducing traffic congestion and environmental pollution but also foster healthier and more sustainable transportation systems for communities.

### Sensitivity analysis

In this section, the impact of changing the parameter values of the mathematical model is tested. The correlation between the parameters and how they affect variables and the objective function when one of them changes values is considered, along with the resulting consequences for the entire system.

**Fig. 7:**
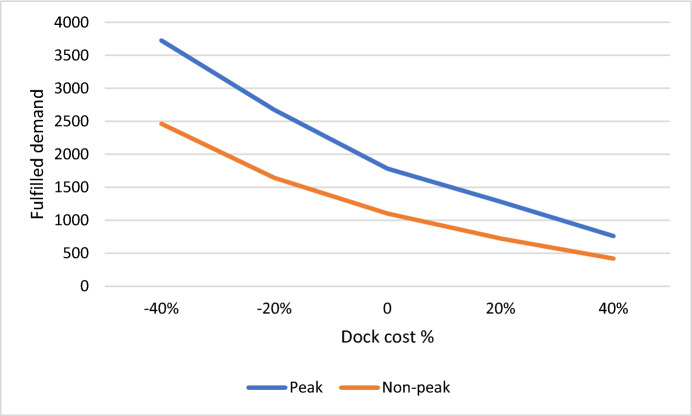
Sensitivity analysis of objective function due to the changes in bikes docks cost

Figure 7 illustrates the impact of altering the cost of bicycle docks, ranging from −40 to + 40%. It is evident from the analysis that as dock costs increase, the fulfilled demand (objective function) decreases. This observation underscores the critical role of cost management in meeting demand efficiently. Moreover, the comparison between peak and non-peak scenarios during the Covid-19 period sheds light on a crucial managerial insight. The peak scenario demonstrates a higher sensitivity to changes in dock costs, as indicated by the steeper slope of its graph compared to the non-peak scenario. This finding emphasizes the need for strategic planning and resource allocation during peak periods to effectively manage demand fluctuations. Additionally, the greater demand volume in the peak scenario results in a higher level of fulfilled demand compared to the non-peak scenario. This highlights the importance of adaptive strategies tailored to different demand scenarios, ensuring optimal utilization of resources and meeting customer needs effectively.

**Fig. 8:**
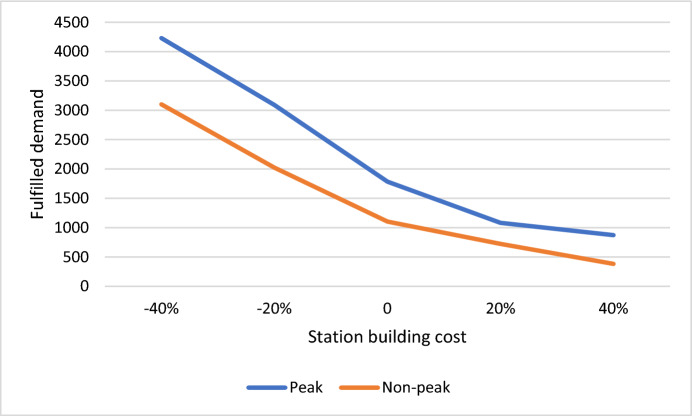
Sensitivity analysis of objective function due to the station building cost

Figure 8 provides insights into the implications of altering the bike station building cost on the fulfilled demand (objective function), ranging from −40 to + 40%. The findings reveal a consistent trend wherein an increase in the station building cost leads to a decrease in the fulfilled demand for both scenarios. Notably, this decrease is more pronounced in the non-peak scenario compared to the peak scenario. This observation underscores the critical role of cost management in optimizing resource allocation and meeting demand effectively. Managers should carefully consider cost implications, particularly during non-peak periods, to mitigate the impact on fulfilled demand. Strategic planning and proactive measures to control building costs can help maintain optimal service levels across different demand scenarios, ensuring customer satisfaction and operational efficiency.

**Fig. 9:**
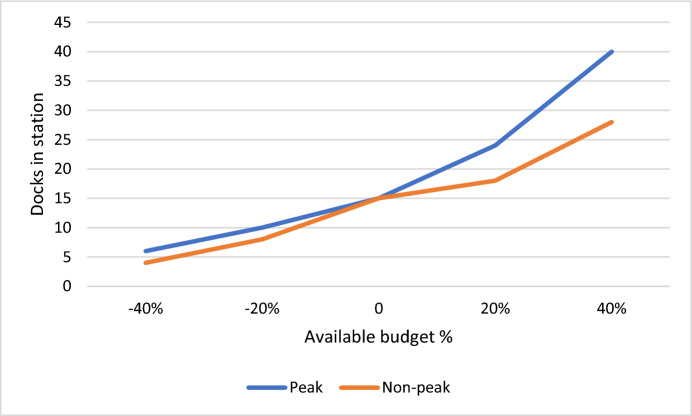
Sensitivity analysis of objective function due to the station building cost

Figure 9 illustrates our examination of the implications of varying the available budget, ranging from −40 to + 40%. It was observed that as the available budget increases, the number of bicycle docks in each station also increases. Furthermore, the slope of the increase in the number of docks in the peak mode is higher than in the non-peak scenario due to the higher volume of demand. This insight emphasizes the importance of budget allocation in meeting demand effectively, particularly during peak periods. Managers should strategically allocate resources to accommodate fluctuating demand levels, ensuring optimal service provision and customer satisfaction.

### Investigation of CPU time of model

**Fig. 10:**
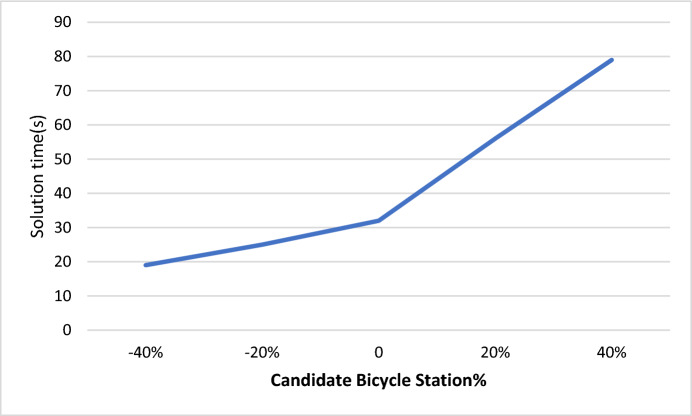
Sensitivity analysis of the taken solution time according to the changes in number of candidate bicycle station

In this section, particular emphasis was placed on studying the effect of changing the value of indices on the time taken to solve the programming model. Figure 10 illustrates the relationship between the change in candidate bicycle station values according to certain percentages and the solution time. It was observed that as the number of candidate bicycle stations increases, the solution time also increases. For example, with a 20% increase in the number of candidate bicycle stations, the solution time is equal to 56 s, whereas with a 20% decrease in the number of candidate bicycle stations, the solution time is equal to 25 s.

**Fig. 11:**
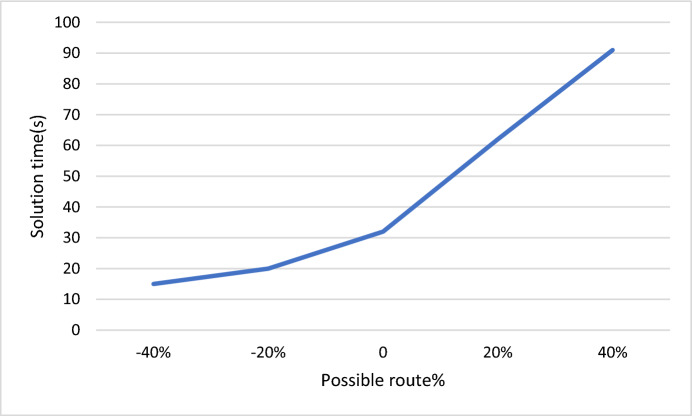
Sensitivity analysis of objective value due to the possible route number

Figure 11 illustrates the effect and relationship between the change in possible route for the origin–destination number according to certain percentages and the solution time. It was observed that as the number of possible paths for the origin–destination number increases, the solution time also increases. For instance, with a 20% increase in the number of possible paths for the origin–destination number, the solution time is equal to 62 s, while with a 20% decrease in candidate bicycle stations, the solution time is equal to 20 s.

## Conclusion

In this paper, a stochastic integer programming model is utilized to optimize network cycle paths for a bike-sharing system, emphasizing the selection of shortest paths to encourage system usage. The research establishes a comprehensive network comprising bike stations, routes, station distances, and connecting pop-up bike lanes. By assessing travel demand under varying conditions and times, the study aims to identify robust solutions. Results indicate that modifying bicycle dock costs influences fulfilled demand, with an observed decrease as dock costs rise. This underscores the pivotal role of cost management in efficiently meeting demand. Notably, during the Covid-19 period, peak scenarios exhibit heightened sensitivity to dock cost adjustments compared to non-peak scenarios, emphasizing the necessity for planning and resource allocation during peak periods. Preparing a bike-sharing system for high demand during peak periods can be both beneficial and challenging. While it ensures that the infrastructure can accommodate sudden surges in usage, promoting better accessibility and user satisfaction during these times, it also requires careful cost management and planning. Likewise, adjustments to bike station building costs impact fulfilled demand, showing decreased demand when building costs increase, particularly noticeable during non-peak periods. Finally, variations in the available budget affect the number of bicycle docks, with an increase in docks per station as the budget increases.

The comparison between scenarios highlights the dynamic nature of infrastructure expansion in response to varying demand levels. During peak periods, infrastructure is expanded to accommodate higher demand, whereas during non-peak periods, a more conservative approach is taken to align with lower demand levels. Also, while both peak and non-peak scenarios aim to optimize infrastructure investments, the peak scenario may prioritize meeting demand even at a higher cost, whereas the non-peak scenario may focus on cost optimization while still maintaining essential services. Overall, the comparison between peak and non-peak scenarios underscores the challenge of balancing accessibility for cyclists with cost considerations in designing and optimizing cycling networks across different demand scenarios. Strategies may vary based on the specific needs and characteristics of each scenario to ensure efficient and sustainable infrastructure development.

Future suggestions based on this research are as follows: By incorporating data such as current bike availability, traffic conditions, and weather patterns, the model can dynamically adjust cycle path recommendations. This approach would enhance the model's adaptability and responsiveness to changing environmental factors. Additionally, incorporating advanced machine learning, metaheuristics techniques and big data analytics into the research methodology could improve the predictive capabilities of the models used. Furthermore, by adjusting bike rental fees based on demand and station availability, the system can incentivize users to redistribute bikes more efficiently across the network. Finally, investigating this alternative by adapting our model to a framework that considers only the spatial radius for demand registration – omitting the explicit consideration of docks – would be a valuable direction for research, particularly in systems where the granularity of dock placement does not significantly impact overall performance or cost-effectiveness.

## Stochastic chance constraint programming

We assume that ~ is the symbol of an uncertain parameter and *n* is the number of objective functions that we have in our mathematical model. Also, we assume that parameters ${a}_{mn}, {h}_{m}, {c}_{nl}$ are defined as stochastic parameter, which are used in Eq. (21) through Eq. (24). Accordingly, the uncertain model is shown below as follows:

21 $$\begin{gathered} \min f_{h} = E\left( {\mathop \sum \limits_{j = 1}^{n} b_{rj}^{\sim } v_{j} \ge u_{i}^{\sim } } \right)\;h = 1, \ldots ,K \hfill \\ i = 1,2, \ldots ,m \hfill \\ \end{gathered}$$

22 $$p\left({\sum }_{j=1}^{n} {g}_{ij}^{\sim }{v}_{j}\ge {u}_{i}^{\sim }\right)\ge {\alpha }_{i}\;i=\text{1,2},\dots ,m$$

23 $$v=\left({v}_{1},\dots ,{v}_{n}\right)$$

24 $$v\ge 0$$

where ${b}_{rj}^{\sim }$  represents the benefit ratio of the $jth$ decision variable in the $rth$ objective function. Additionally, other parameters such as ${g}_{ij}^{\sim }$ represent the technology coefficient of the $nth$ decision variable in the $ith$ constraint, ${u}_{i}^{\sim }$ is the right-hand side of the $mth$ constraint, and ${v}_{j}$ is also a decision variable.

The value of ${\alpha }_{i}$ represents the maximum allowable probability of violating the constraint. Additionally, $p(.)$ represents the probability of this occurrence. So, the stochastic model is as shown in (25–28).

25 $$\begin{gathered} \min f_{h} = E\left( {\mathop \sum \limits_{j = 1}^{n} b_{rj}^{\sim } v_{j} \ge u_{i}^{\sim } } \right)\;h = 1, \ldots ,H \hfill \\ i = 1,2, \ldots ,m \hfill \\ \end{gathered}$$

26 $$p\left( {\sum\limits_{{j = 1}}^{n} {g_{{ij}}^{\sim } v_{j} \ge u_{i}^{\sim } } } \right) \ge \alpha _{i} \;i = {\text{1,2}}, \ldots ,m$$

27 $$v=\left({v}_{1},\dots ,{v}_{n}\right)$$

28 $$v\ge 0$$

Here, it is assumed that the uncertain parameters ${g}_{ij}^{\sim }$ and ${u}_{i}^{\sim }$ have a certain average and standard deviation; it is assumed that:

29 $$R_{i}^{\sim } \left( v \right) = \sum\limits_{{j = 1}}^{n} {g_{{ij}}^{\sim } v_{j} - u_{i}^{\sim } } \;i = {\text{1,2}}, \ldots ,m$$

Therefore, ${R}_{i}^{\sim }\left(v\right)$ is a random variable with average $E({R}_{i}^{\sim }\left(v)\right)$ and variance $Var({R}_{i}^{\sim }\left(v)\right)$. Consequently, the Eq. (26) is expressed as Eq. (30):

30 $$p({R}_{i}^{\sim }\left(v\right)\ge 0))\ge {\alpha }_{i}\;i=\text{1,2},\dots ,m$$

According to Eqs. (21)–(24), the variable ${R}_{i}^{\sim }\left(v\right)$ will also follow a standard normal distribution.

31 $$1 - p(R_{i}^{\sim } \left( v \right)) \le 0) \ge \alpha _{i} \quad i = {\text{1,2}}, \ldots ,m$$

32 $$1 - p\left( {\frac{{R_{i}^{\sim } \left( v \right) - E\left( {R_{i}^{\sim } \left( v \right)} \right)}}{{\sqrt {Var\left( {R_{i}^{\sim } \left( v \right)} \right)} }} \le \frac{{ - E\left( {R_{i}^{\sim } \left( v \right)} \right)}}{{\sqrt {Var(} R_{i}^{\sim } \left( v \right))}}} \right) \ge \alpha _{i} \quad i = {\text{1,2}}, \ldots ,m$$

33 $$1-p(z(v))\le \frac{-E\left({R}_{i}^{\sim }\left(v\right)\right)}{\sqrt{Var(}{R}_{i}^{\sim }\left(v\right))}))\ge {\alpha }_{i}\quad i=\text{1,2},\dots ,m$$

34 $$p(z(v))\le \frac{-E({R}_{i}^{\sim }\left(v\right))}{\sqrt{Var(}{R}_{i}^{\sim }\left(v\right))}))\le {1-\alpha }_{i}$$

In this equation, $z\left(v\right)$ is the standard normal variable, and based on the concept of the cumulative density function, we have:

35 $$\frac{-E({R}_{i}^{\sim }\left(v\right))}{\sqrt{Var(}{R}_{i}^{\sim }\left(v\right))})\le {\varphi }^{-1}{(1-\alpha }_{i})$$

36 $$E\left( {R_{i}^{\sim } \left( v \right)\varphi ^{{ - 1}} (1 - \alpha _{i} )\sqrt {Var(} R_{i}^{\sim } \left( v \right)} \right) \ge 0$$

Therefore, by placing ${R}_{i}^{\sim }\left(v\right)={\sum }_{j=1}^{n}{g}_{ij}^{\sim }.{v}_{j}-{u}_{i}^{\sim }$, we have:

37 $$E\left( {\sum\limits_{{j = 1}}^{n} {g_{{ij}}^{\sim } v_{j} - u_{i}^{\sim } - \varphi ^{{ - 1}} (1 - \alpha _{i} )\sqrt {Var} } } \right)\sum\limits_{{j = 1}}^{n} {g_{{ij}}^{\sim } v_{j} - u_{i}^{\sim } \ge 0}$$

To transform the stochastic model to a deterministic one, we first assume that ${w}_{hi}^{*}$ is the maximum observable value of the objective function $h$: ${w}_{hi}^{*} =Max {w}_{hi}^{\sim }$

The value of ${w}_{hi}^{*}$ at the confidence level $\alpha \%$ is as follows:

38 $${w}_{hi}^{*}=E\left({w}_{hi}^{\sim }\right)+{z}_{\alpha }.\sqrt{var(}{w}_{hi}^{\sim })$$

In this case, $E\left({b}_{rj}^{\sim }\right)$ and $\sqrt{var(}{b}_{rj}^{\sim })$ represent the mean and standard deviation, respectively, of the random variable ${b}_{rj}^{\sim }$. Meanwhile, Eq. (38) determines the upper bound of the normal random variable ${b}_{rj}^{\sim }$ at a confidence level of $\alpha \%$. By substituting ${b}_{rj}^{*}$ into all the objective functions of Eq. (25) and solving $r$ separate single-objective problems, we obtain the following outcome: ${f}_{r}^{+}=Max{b}_{rj}^{*}{v}_{j}$

Conversely, the single-objective optimization model will be solved $k$ times using Eqs. (39)–(41) to determine the upper bound solution for the objective functions at an α% confidence level.

39 $${f}_{r}^{+}=Max{b}_{rj}^{*}{v}_{j}$$

40 $$v\in S$$

41 $$v\ge 0$$

The values of ${f}_{r}^{+} r=\text{1,2},\dots ,R$ represent the upper limits of the objective functions. Consequently, Eqs. (42) and (43) are expressed as follows:

42 $${\sum_{j=1}{b}_{rj}^{*}{v}_{j}}\le {f}_{r}^{+}$$

43 $$p({b}_{rj}^{*}{v}_{j}\le {f}_{r}^{+})\ge {\alpha }_{r}$$

The definition of the variable ${G}_{r}^{\sim }\left(v\right)$ is as follows:

44 $$G_{r}^{\sim } \left( v \right) = \sum\limits_{{j = 1}}^{n} {b_{{rj}}^{*} v_{j} - f_{r}^{ + } }$$

Therefore, we rewrite Eq. (43) as Eq. (45):

45 $$p({G}_{r}^{\sim }\left(v\right))\le 0)\ge {\alpha }_{r}$$

Consequently, the final value for Eq. (45) is expressed through Eqs. (46–48a).

46 $$p(\frac{{G}_{r}^{\sim }\left(v\right)-E\left({G}_{i}^{\sim }\left(v\right)\right)}{\sqrt{Var(}{G}_{r}^{\sim }\left(v\right))}\le \frac{-E({G}_{r}^{\sim }\left(v\right)}{\sqrt{Var(}{G}_{r}^{\sim }\left(v\right))}))\ge {\alpha }_{r}$$

47 $$p(\frac{-E\left({G}_{i}^{\sim }\left(v\right)\right)}{\sqrt{Var(}{G}_{r}^{\sim }\left(v\right))}\ge {\varnothing }^{-1}({\alpha }_{r})$$

48 $$E({G}_{r}^{\sim }\left(v\right)+{\varphi }^{-1}{(\alpha }_{r})\sqrt{Var(}{G}_{r}^{\sim }\left(v\right))\le 0$$

By substituting the values ${G}_{i}^{\sim }\left(v\right)={\sum }_{j=1}^{n}{b}_{rj}^{*}{v}_{j}-{f}_{r}^{+}$ into Eq. (25), the upper bound for the values of the objective functions at the confidence level of α% is given by Eq. (49).

49 $$E\left( {\sum\limits_{{j = 1}}^{n} {b_{{rj}}^{*} v_{j} - f_{k}^{ + } } } \right) + \varphi ^{{ - 1}} (\alpha _{r} )\sqrt {Var} \left( {\sum\limits_{{j = 1}}^{n} {b_{{rj}}^{*} v_{j} - f_{k}^{ + } } } \right) \le 0$$

We also assume that ${b}_{rj}^{*}$ represents the minimum value in the minimization functions. The value of ${b}_{rj}^{*}$ can be determined based on the definition of the normal variable at the α% level, as shown in Eq. (51).

50 $${b}_{rj}^{*}=Min {b}_{rj}^{\sim }$$

51 $${b}_{rj}^{*}=E\left({b}_{rj}^{\sim }\right)-{z}_{\alpha }.\sqrt{var(}{b}_{rj}^{\sim })$$

$E\left({b}_{rj}^{\sim }\right)$ represent the mean and $\sqrt{var(}{b}_{rj}^{\sim })$ standard deviation, respectively, of the random variable ${b}_{rj}^{\sim }$ at the α% confidence level. By substituting ${b}_{rj}^{*}$ into all objective functions of Eq. (25) and solving $r$ separate single-objective problems, we obtain: $f_{r}^{ - } = Min\sum\nolimits_{{j = 1}}^{n} {b_{{rj}}^{\sim } } v_{j}$

Conversely, $r$ single-objective optimization model will be solved using Eqs. (52)–(54) to determine the upper bound solution for the objective functions at the α% confidence level.

52 $$f_{r}^{ - } = \sum\limits_{{j = 1}}^{n} {Minb_{{rj}}^{*} v_{j} }$$

53 $$v\in S$$

54 $$v\ge 0$$

The values of ${f}_{r}^{-} r=\text{1,2},\dots ,R$ represent the upper bounds of the objective functions. Consequently, Eqs. (55) and (56) can be expressed as follows:

55 $${\sum_{j=1}{b}_{rj}^{*}{v}_{j}}\ge {f}_{r}^{-}$$

56 $${p(b}_{rj}^{*}{v}_{j}\ge {f}_{r}^{-})\ge {\alpha }_{r}$$

Next, the definition of the variable ${G}_{r}^{\sim }\left(v\right)$ is as follows:

57 $$G_{r}^{\sim } \left( v \right) = \sum\limits_{{j = 1}}^{n} {b_{{rj}}^{*} v_{j} - f_{r}^{ - } }$$

Therefore, Eq. (56) can be written as Eq. (58):

58 $$p\left({G}_{r}^{\sim }\left(v\right)\ge 0\right)\ge {\alpha }_{r} r=1,\dots ,R$$

It is known that ${G}_{r}^{\sim }\left(v\right)$ follows a normal distribution with a mean of $E\left({G}_{r}^{\sim }\left(v\right)\right)$ and a standard deviation of $Var{(G}_{r}^{\sim }\left(v\right))$. Therefore, the exact expression for Eq. 61 (58) can be represented by Eqs. (59–63):

59 $$p\left( {\frac{{G_{r}^{\sim } \left( v \right) - E\left( {G_{r}^{\sim } \left( v \right)} \right)}}{{\sqrt {Var(} G_{r}^{\sim } \left( v \right))}} \ge \frac{{ - E(G_{r}^{\sim } \left( v \right))}}{{\sqrt {Var(} G_{r}^{\sim } \left( v \right))}}} \right) \ge \alpha _{r}$$

60 $$1 - p\left( {\frac{{G_{r}^{\sim } \left( v \right) - E\left( {G_{r}^{\sim } \left( v \right)} \right)}}{{\sqrt {Var(} G_{r}^{\sim } \left( v \right))}} \le \frac{{ - E(G_{r}^{\sim } \left( v \right))}}{{\sqrt {Var(} G_{r}^{\sim } \left( v \right))}}} \right) \ge \alpha _{r}$$

61 $$p\left( {\frac{{G_{r}^{\sim } \left( v \right) - E\left( {G_{r}^{\sim } \left( v \right)} \right)}}{{\sqrt {Var(} G_{r}^{\sim } \left( v \right))}} \le \frac{{ - E(G_{r}^{\sim } \left( v \right))}}{{\sqrt {Var(} G_{r}^{\sim } \left( v \right))}}} \right) \le \alpha _{r}$$

62 $$\frac{-E\left({G}_{r}^{\sim }\left(v\right)\right)}{\sqrt{Var(}{G}_{r}^{\sim }\left(v\right))}\le {\varnothing }^{-1}\left({\alpha }_{r}\right)$$

63 $$E({G}_{r}^{\sim }\left(v\right)+{\varphi }^{-1}{(\alpha }_{r})\sqrt{Var(}{G}_{r}^{\sim }\left(v\right)\ge 0$$

Substituting the values ${G}_{r}^{\sim }\left(v\right)={\sum\limits }_{j=1}^{n}{b}_{rj}^{*}{v}_{j}-{f}_{r}^{-}$ into Eq. (63), the lower bound of the objective function values at a confidence level of $\alpha$% is given by Eq. (64):

64 $$E\left( {\sum\limits_{{j = 1}}^{n} {b_{{rj}}^{*} v_{j} - f_{r}^{ - } } } \right) + \varphi ^{{ - 1}} (\alpha _{r} )\sqrt {Var} \left( {\sum\limits_{{j = 1}}^{n} {b_{{rj}}^{*} v_{j} - f_{r}^{ - } } } \right) \ge 0$$

Based on Eqs. (37), (49) and (64), the multi-objective model of the chance constraint can be defined as a deterministic multi-objective model at the $\alpha$% level. The next problem is the multi-objective nature of the model. This problem can be solved with the Goal programming approach. This method has two kinds of constraints: system constraints and objective constraints. For objective constraints, deviation variables $d_{k}^{ + } ,d_{k}^{ - }$ are introduced. To address both maximization and minimization models, the objective is to minimize these deviations. The ideal planning is represented by Eqs. (65) through (72).

65 $$Min\sum\limits_{{k = 1}}^{n} {\left[ {w_{r} .\frac{{d_{r}^{ + } + d_{r}^{ - } }}{{z_{r}^{ - } - z_{r}^{ + } }}} \right]}$$

66 $$\sum\limits_{{j = 1}}^{n} {g_{{ij}} v_{j} } \ge u_{i}$$

67 $${z}_{r}\left(v\right)+\left({d}_{r}^{-}-{d}_{r}^{+}\right)={z}_{r}^{*}$$

68 $${d}_{r}^{-}.{d}_{r}^{+}=0$$

69 $${d}_{r}^{-},{d}_{r}^{+}\ge 0$$

70 $$v=({v}_{1},\dots ,{v}_{n})$$

71 $$v\in S$$

72 $$v\ge 0$$

The values ${d}_{r}^{+} and {d}_{r}^{-}$ represent the positive and negative deviations of the function $r$, respectively. ${w}_{\text{r}}$ denotes the weighted importance of the objective function $r$. The value ${z}_{r}^{-}$ is the negative ideal for the objective function $r$, derived by solving the single-objective minimization model $r$ times, as shown in Eqs. (52)–(56). Similarly, ${z}_{k}^{+}$ represents the positive ideal for the objective function $r$, obtained by solving the single-objective maximization model $r$ times, also as given in Eqs. (52)–(56). The value ${z}_{k}^{*}$ corresponds to the final value of the objective function $r$.

Consequently, constraint (9) will be transformed into their deterministic form as (73).

73 $${R}_{et} (\xi )\le (E\left({T}_{et} \left(\xi \right)\right)+{\varphi }^{-1}\left(1-{\alpha }_{i}\right)\sqrt{var\left({T}_{et} \left(\xi \right)\right)} \forall e\in E,\forall t\in T,\forall \xi \in\Omega$$

## Appendix B

In this appendix, we present the most important parameters of the model in Tables

**Table 4:** Bikes station building cost

${C}_{a}$	Sharing Station Location	Building cost (Euro)
B1	2, Pazmanitengasse, Vienna	40,000
B2	2, Alliiertestraße, Vienna	35,000
B3	3, Rechte Bahngasse, Vienna	30,000
B4	3, Schützengasse, Vienna	30,000
B5	4, Graf-Starhemberg-Gasse, Vienna	25,000
B6	4, Schaumburgergasse, Vienna	25,000
B7	4, Große Neugasse, Vienna	25,000
B8	4/5, Kettenbrückengasse, Vienna	20,000
B9	5, Rüdigergasse, Vienna	20,000
B10	7, Kandlgasse, Vienna	15,000
B11	7, Hermanngasse, Vienna	15,000
B12	7, Zollergasse, Vienna	15,000
B13	8, Florianigasse, Vienna	10,000
B14	9, Sobieskigasse, Vienna	10,000
B15	10, Fernkorngasse, Vienna	10,000
B16	14/15, Meiselstraße, Vienna	10,000
B17	15, Rosinagasse, Vienna	10,000
B18	15, Gasgasse, Vienna	10,000
B19	15, Zwölfergasse, Vienna	10,000
B20	16, Hasnerstraße, Vienna	5000
B21	17, Kalvarienberggasse, Vienna	5000
B22	18, Schopenhauerstraße, Vienna	5000
B23	20, Brigittenauer Sporn, Vienna	5000

^4^,

**Table 5:** Distances between sharing stations (KM)

${d}_{ab}$	Sharing Station Location	B1	B2	B3	B4	B5	B6	B7	B8	B9	B10	B11	B12	B13	B14	B15	B16	B17	B18	B19	B20	B21	B22	B23
B1	2, Pazmanitengasse, Vienna	0	0	1,9	2,8	3,4	3,4	3,4	3,3	3,6	3,5	3,5	3,4	2,4	2,4	4,7	5,8	4,8	4,7	4,9	3,9	3,9	2,9	4,4
B2	2, Alliiertestraße, Vienna	0	0	1,9	2,8	3,4	3,4	3,4	3,3	3,6	3,5	3,5	3,4	2,4	2,4	4,7	5,8	4,8	4,7	4,9	3,9	3,9	2,9	4,4
B3	3, Rechte Bahngasse, Vienna	2	1,9	0	0,9	1,7	1,8	1,8	1,9	2,2	2,9	2,8	2,5	2,5	3,5	3	5,2	4	3,8	4	3,7	4,2	3,7	6,2
B4	3, Schützengasse, Vienna	3	2,8	0,9	0	1,5	1,5	1,6	1,9	2,2	3,3	3,1	2,7	3,1	4,4	2,4	5,4	4,1	3,9	4,2	4,1	4,8	4,5	7,1
B5	4, Graf-Starhemberg-Gasse, Vienna	3	3,4	1,7	1,5	0	0,1	0,1	0,6	0,8	2,1	1,9	1,5	2,5	4,1	1,4	4,1	2,7	2,5	2,8	3,1	3,9	3,9	7,4
B6	4, Schaumburgergasse, Vienna	3	3,4	1,8	1,5	0,1	0	0,1	0,6	0,8	2,1	1,9	1,5	2,5	4,1	1,3	4	2,7	2,5	2,7	3	3,9	4	7,4
B7	4, Große Neugasse, Vienna	3	3,4	1,8	1,6	0,1	0,1	0	0,5	0,7	2	1,8	1,4	2,5	4	1,3	3,9	2,6	2,4	2,6	2,9	3,8	3,9	7,4
B8	4/5, Kettenbrückengasse, Vienna	3	3,3	1,9	1,9	0,6	0,6	0,5	0	0,3	1,6	1,3	1	2,1	3,7	1,6	3,6	2,2	2	2,3	2,5	3,3	3,5	7,1
B9	5, Rüdigergasse, Vienna	4	3,6	2,2	2,2	0,8	0,8	0,7	0,3	0	1,4	1,1	0,8	2,1	3,7	1,6	3,2	1,9	1,7	2	2,3	3,2	3,5	7,3
B10	7, Kandlgasse, Vienna	4	3,5	2,9	3,3	2,1	2,1	2	1,6	1,4	0	0,2	0,6	1,3	2,8	2,9	2,3	1,3	1,2	1,3	0,9	1,9	2,4	6,5
B11	7, Hermanngasse, Vienna	4	3,5	2,8	3,1	1,9	1,9	1,8	1,3	1,1	0,2	0	0,4	1,3	2,9	2,7	2,4	1,3	1,2	1,4	1,2	2,1	2,5	6,6
B12	7, Zollergasse, Vienna	3	3,4	2,5	2,7	1,5	1,5	1,4	1	0,8	0,6	0,4	0	1,5	3,1	2,4	2,7	1,5	1,3	1,6	1,5	2,4	2,8	6,7
B13	8, Florianigasse, Vienna	2	2,4	2,5	3,1	2,5	2,5	2,5	2,1	2,1	1,3	1,3	1,5	0	1,6	3,7	3,3	2,6	2,5	2,6	1,5	1,7	1,4	5,3
B14	9, Sobieskigasse, Vienna	2	2,4	3,5	4,4	4,1	4,1	4	3,7	3,7	2,8	2,9	3,1	1,6	0	5,3	4,3	4	3,9	3,9	2,5	1,9	0,7	3,7
B15	10, Fernkorngasse, Vienna	5	4,7	3	2,4	1,4	1,3	1,3	1,6	1,6	2,9	2,7	2,4	3,7	5,3	0	4,2	2,9	2,7	3	3,8	4,8	5,1	8,7
B16	14/15, Meiselstraße, Vienna	6	5,8	5,2	5,4	4,1	4	3,9	3,6	3,2	2,3	2,4	2,7	3,3	4,3	4,2	0	1,4	1,6	1,3	1,9	2,5	3,7	7,9
B17	15, Rosinagasse, Vienna	5	4,8	4	4,1	2,7	2,7	2,6	2,2	1,9	1,3	1,3	1,5	2,6	4	2,9	1,4	0	0,2	0,1	1,5	2,5	3,4	7,6
B18	15, Gasgasse, Vienna	5	4,7	3,8	3,9	2,5	2,5	2,4	2	1,7	1,2	1,2	1,3	2,5	3,9	2,7	1,6	0,2	0	0,3	1,5	2,6	3,4	7,6
B19	15, Zwölfergasse, Vienna	5	4,9	4	4,2	2,8	2,7	2,6	2,3	2	1,3	1,4	1,6	2,6	3,9	3	1,3	0,1	0,3	0	1,5	2,5	3,4	7,6
B20	16, Hasnerstraße, Vienna	4	3,9	3,7	4,1	3,1	3	2,9	2,5	2,3	0,9	1,2	1,5	1,5	2,5	3,8	1,9	1,5	1,5	1,5	0	1,1	2	6,2
B21	17, Kalvarienberggasse, Vienna	4	3,9	4,2	4,8	3,9	3,9	3,8	3,3	3,2	1,9	2,1	2,4	1,7	1,9	4,8	2,5	2,5	2,6	2,5	1,1	0	1,2	5,3
B22	18, Schopenhauerstraße, Vienna	3	2,9	3,7	4,5	3,9	4	3,9	3,5	3,5	2,4	2,5	2,8	1,4	0,7	5,1	3,7	3,4	3,4	3,4	2	1,2	0	4,2
B23	20, Brigittenauer Sporn, Vienna	4	4,4	6,2	7,1	7,4	7,4	7,4	7,1	7,3	6,5	6,6	6,7	5,3	3,7	8,7	7,9	7,6	7,6	7,6	6,2	5,3	4,2	0

^5^ and

**Table 6:** Bike paths building cost for link $(a,b)$

${C}_{ab}$	B1	B2	B3	B4	B5	B6	B7	B8	B9	B10	B11	B12	B13	B14	B15	B16	B17	B18	B19	B20	B21	B22	B23
B1	0	500	1000	1500	2000	2000	2000	2000	2000	2000	2000	2000	1500	1500	2500	3000	2500	2500	2500	2000	2000	1500	2500
B2	500	0	1000	1500	2000	2000	2000	2000	2000	2000	2000	2000	1500	1500	2500	3000	2500	2500	2500	2000	2000	1500	2500
B3	1000	1000	0	500	1000	1000	1000	1000	1500	2000	2000	1500	1500	2000	2000	3000	2500	2500	2500	2000	2500	2000	3500
B4	1500	1500	500	0	1000	1000	1000	1000	1500	2000	2000	1500	2000	2500	1500	3000	2500	2500	2500	2500	3000	3000	4000
B5	2000	2000	1000	1000	0	500	500	500	1000	1500	1500	1000	1500	2500	1000	2500	2000	2000	2000	2000	2500	2500	4000
B6	2000	2000	1000	1000	500	0	500	500	1000	1500	1500	1000	1500	2500	1000	2500	2000	2000	2000	2000	2500	2500	4000
B7	2000	2000	1000	1000	500	500	0	500	1000	1500	1500	1000	1500	2500	1000	2500	2000	2000	2000	2000	2500	2500	4000
B8	2000	2000	1000	1000	500	500	500	0	500	1500	1500	1000	1500	2500	1500	2500	2000	2000	2000	2000	2500	2500	4000
B9	2000	2000	1500	1500	1000	1000	1000	500	0	1500	1500	1000	1500	2500	1500	2500	2000	2000	2000	2000	2500	2500	4000
B10	2000	2000	2000	2000	1500	1500	1500	1500	1500	0	500	1000	1000	2000	2500	2000	1500	1500	1500	1000	1500	2000	3500
B11	2000	2000	2000	2000	1500	1500	1500	1500	1500	500	0	500	1000	2000	2500	2000	1500	1500	1500	1500	2000	2000	3500
B12	2000	2000	1500	1500	1000	1000	1000	1000	1000	1000	500	0	1000	2000	2500	2000	1500	1500	1500	1500	2000	2000	3500
B13	1500	1500	1500	2000	1500	1500	1500	1500	1500	1000	1000	1000	0	1000	2500	2500	2000	2000	2000	1500	1500	1000	3000
B14	1500	1500	2000	2500	2500	2500	2500	2500	2500	2000	2000	2000	1000	0	3000	2500	2500	2500	2500	1500	1000	500	2500
B15	2500	2500	2000	1500	1000	1000	1000	1500	1500	2500	2000	2000	2500	3000	0	2500	2000	2000	2000	2500	3000	3000	4000
B16	3000	3000	3000	3000	2500	2500	2500	2500	2500	2000	2000	2000	2500	2500	2500	0	1000	1000	1000	1500	2000	2500	4000
B17	2500	2500	2500	2500	2000	2000	2000	2000	2000	1500	1500	1500	2000	2500	2000	1000	0	500	500	1500	2000	2500	4000
B18	2500	2500	2500	2500	2000	2000	2000	2000	2000	1500	1500	1500	2000	2500	2000	1000	500	0	500	1500	2000	2500	4000
B19	2500	2500	2500	2500	2000	2000	2000	2000	2000	1500	1500	1500	2000	2500	2000	1000	500	500	0	2000	3000	3000	2500
B20	2000	2000	2000	2500	2000	2000	2000	2000	2000	1000	1500	1500	1500	1500	2500	1500	1500	1500	2000	0	2500	4000	3500
B21	2000	2000	2500	3000	2500	2500	2500	2500	2500	1500	2000	2000	1500	1000	3000	2000	2000	2000	3000	2500	0	3000	4000
B22	1500	1500	2000	3000	2500	2500	2500	2500	2500	2000	2000	2000	1000	500	3000	2500	2500	2500	3000	4000	3000	0	2000
B23	2500	2500	3500	4000	4000	4000	4000	4000	4000	3500	3500	3500	3000	2500	4000	4000	4000	4000	2500	3500	4000	2000	0

^6^. Additionally, we consider the maximum number of bicycle docks at each station, which is set to 30.

## References

[CR1] Becker S, von Schneidemesser D, Caseiro A, Götting K, Schmitz S, von Schneidemesser E (2022) Pop-up cycling infrastructure as a niche innovation for sustainable transportation in European cities: an inter-and transdisciplinary case study of Berlin. Sustain Cities Soc 87:104168

[CR01] Charnes A, Cooper WW (1959) Chance-constrained programming. Manag Sci 6(1):73–79

[CR2] Chen Y, Sun X, Deveci M, Coffman DM (2022) The impact of the COVID-19 pandemic on the behaviour of bike sharing users. Sustain Cities Soc 84:104003 35756367 10.1016/j.scs.2022.104003 PMC9212929

[CR3] Chen Q, Fu C, Zhu N, Ma S, He QC (2023) A target-based optimization model for bike-sharing systems: from the perspective of service efficiency and equity. Transp Res Part B: Methodol 167:235–260

[CR4] Cheng R, Zhong S, Wang Z, Nielsen OA, Jiang Y (2022) A hyper-heuristic approach to the strategic planning of bike-sharing infrastructure. Comput Ind Eng 173:108704

[CR5] Chibwe J, Heydari S, Imani AF, Scurtu A (2021) An exploratory analysis of the trend in the demand for the London bike-sharing system: from London olympics to Covid-19 pandemic. Sustain Cities Soc 69:102871

[CR6] Dechant HE (2013) 17.06.2013. Bike sharing systeme. Accessed from. https://bit.ly/2N75uXs.

[CR7] Fishman E, Washington S, Haworth N (2012) Barriers and facilitators to public bicycle scheme use: a qualitative approach. Transp Res F: Traffic Psychol Behav 15(6):686–698

[CR8] Frey H, Laa B, Leth U (2023) Pop-up bike lanes and temporary shared spaces in Vienna during the COVID-19 pandemic. Cycling through the pandemic: Tactical urbanism and the implementation of pop-up bike lanes in the time of COVID-19. Springer International Publishing, Cham, pp 139–167

[CR9] Fu C, Zhu N, Ma S, Liu R (2022) A two-stage robust approach to integrated station location and rebalancing vehicle service design in bike-sharing systems. Eur J Oper Res 298(3):915–938

[CR10] Guo Y, Li J, Xiao L, Allaoui H, Choudhary A, Zhang L (2024) Efficient inventory routing for Bike-sharing systems: a combinatorial reinforcement learning framework. Transp Res Part E: Logist Transp Rev 182:103415

[CR11] Hintermann B, Schoeman B, Molloy J, Schatzmann T, Tchervenkov C, Axhausen KW (2023) The impact of COVID-19 on mobility choices in Switzerland. Transp Res Part a: Policy Pract 169:103582 36685312 10.1016/j.tra.2023.103582 PMC9841083

[CR12] Hua M, Chen X, Chen J, Jiang Y (2022) Minimizing fleet size and improving vehicle allocation of shared mobility under future uncertainty: a case study of bike sharing. J Clean Prod 370:133434

[CR13] Jiang R, Guan Y (2016) Data-driven chance constrained stochastic program. Math Program 158(1–2):291–327

[CR14] Jiménez E, Soriguera F (2024) Optimization of bike-sharing repositioning operations: a reactive real-time approach. EURO J Transp Logist 13:100138

[CR15] Jin ST, Sui DZ (2024) Bikesharing and equity: a nationwide study of bikesharing accessibility in the US. Transp Res Part a: Policy Pract 181:103983

[CR16] Jin JG, Nieto H, Lu L (2020) Robust bike-sharing stations allocation and path network design: a two-stage stochastic programming model. Transp Lett 12(10):682–691

[CR17] Jobe J, Griffin GP (2021) Bike share responses to COVID-19. Transp Res Interdiscip Perspect 10:100353 36844003 10.1016/j.trip.2021.100353 PMC9940611

[CR18] Kamran MA, Kia R, Goodarzian F, Ghasemi P (2023) A new vaccine supply chain network under COVID-19 conditions considering system dynamic: artificial intelligence algorithms. Socioecon Plann Sci 85:101378 35966449 10.1016/j.seps.2022.101378 PMC9359548

[CR19] Karatas M, Erişkin L, Bozkaya E (2022) Transportation and location planning during epidemics/pandemics: emerging problems and solution approaches. IEEE Trans Intell Transp Syst 23(12):25139–25156

[CR20] Kim M, Cho GH (2022) Examining the causal relationship between bike-share and public transit in response to the COVID-19 pandemic. Cities 131:104024 36211221 10.1016/j.cities.2022.104024 PMC9533677

[CR21] Peláez-Rodríguez C, Pérez-Aracil J, Fister D, Torres-López R, Salcedo-Sanz S (2024) Bike sharing and cable car demand forecasting using machine learning and deep learning multivariate time series approaches. Expert Syst Appl 238:122264

[CR22] Rahimi E, Shabanpour R, Shamshiripour A, Mohammadian AK (2021) Perceived risk of using shared mobility services during the COVID-19 pandemic. Transp Res F: Traffic Psychol Behav 81:271–281 10.1016/j.trf.2021.06.012 PMC9761113 36567796

[CR23] Reza-Pour F, Khalili-Damghani K (2017) A new stochastic time-cost-quality trade-off project scheduling problem considering multiple-execution modes, preemption, and generalized precedence relations. Ind Eng Manag Syst 16(3):271–287

[CR24] Schulte-Fischedick M, Shan Y, Hubacek K (2021) Implications of COVID-19 lockdowns on surface passenger mobility and related CO_2_ emission changes in Europe. Appl Energy 300:117396 34305265 10.1016/j.apenergy.2021.117396 PMC8278838

[CR25] Song J, Li B, Szeto WY, Zhan X (2024) A station location design problem in a bike-sharing system with both conventional and electric shared bikes considering bike users’ roaming delay costs. Transp Res Part E: Logist Transp Rev 181:103350

[CR26] Teixeira JF, Lopes M (2020) The link between bike sharing and subway use during the COVID-19 pandemic: the case-study of New York’s Citi Bike. Transp Res Interdiscip Perspect 6:100166 34173457 10.1016/j.trip.2020.100166 PMC7345406

[CR27] Teixeira JF, Silva C, eSá FM (2021) The motivations for using bike sharing during the COVID-19 pandemic: insights from Lisbon. Transp Res Part F: Traffic Psychol Behav 82:378–399 34602849 10.1016/j.trf.2021.09.016 PMC8479539

[CR28] Teixeira JF, Silva C, Moura F, e Sá, (2023) Potential of bike sharing during disruptive public health crises: a review of COVID-19 impacts. Transp Res Rec: J Transp Res Board. 10.1177/03611981231160537

[CR29] Thomas MM, Verma A, Mayakuntla SK, Chandra A (2024) A novel simulation based approach for user-based redistribution in bike-sharing system. Simul Model Pract Theory 131:102871

[CR30] Walker A, Kwon S (2024) Risk-averse two-stage stochastic programming for the inventory rebalancing of bike-sharing systems. Int Trans Oper Res 31(2):749–779

[CR31] Wang X, Zheng S, Wang L, Han S, Liu L (2023) Multi-objective optimal scheduling model for shared bikes based on spatiotemporal big data. J Clean Prod 421:138362

[CR32] Xu SJ, Chow JY (2020) A longitudinal study of bike infrastructure impact on bikesharing system performance in New York City. Int J Sustain Transp 14(11):886–902

